# Corsair rotational bougie technique for facilitating balloon passage through a total occlusion lesion: a case report of balloon pulmonary angioplasty

**DOI:** 10.1093/ehjcr/ytad040

**Published:** 2023-01-24

**Authors:** Hiroto Tamura, Yu Taniguchi, Shinobu Hosokawa, Koichi Kishi

**Affiliations:** Division of Cardiology, Tokushima Red Cross Hospital, 103 Irinokuchi, Komatsushima-cho, Komatsushima, Tokushima 773-8502, Japan; Division of Cardiovascular Medicine, Department of Internal Medicine, Kobe University Graduate School of Medicine, 7-5-2 Kusunoki-cho, Chuo-ku, Kobe 650-0017, Japan; Division of Cardiology, Tokushima Red Cross Hospital, 103 Irinokuchi, Komatsushima-cho, Komatsushima, Tokushima 773-8502, Japan; Division of Cardiology, Tokushima Red Cross Hospital, 103 Irinokuchi, Komatsushima-cho, Komatsushima, Tokushima 773-8502, Japan

**Keywords:** Chronic thromboembolic pulmonary hypertension, Balloon pulmonary angioplasty, Total occlusion lesion, Corsair rotational bougie technique

## Abstract

A 77-year-old man presented with dyspnoea and was diagnosed with chronic thromboembolic pulmonary hypertension. Balloon pulmonary angioplasty was attempted; however, the balloons could not be advanced to the total occlusion lesion in the right A3 segment. The obstruction was overcome using a microcatheter Corsair (AsahiKASEI). This technique may be useful in managing a total occlusion lesion.

## Case description

**Figure 1:**
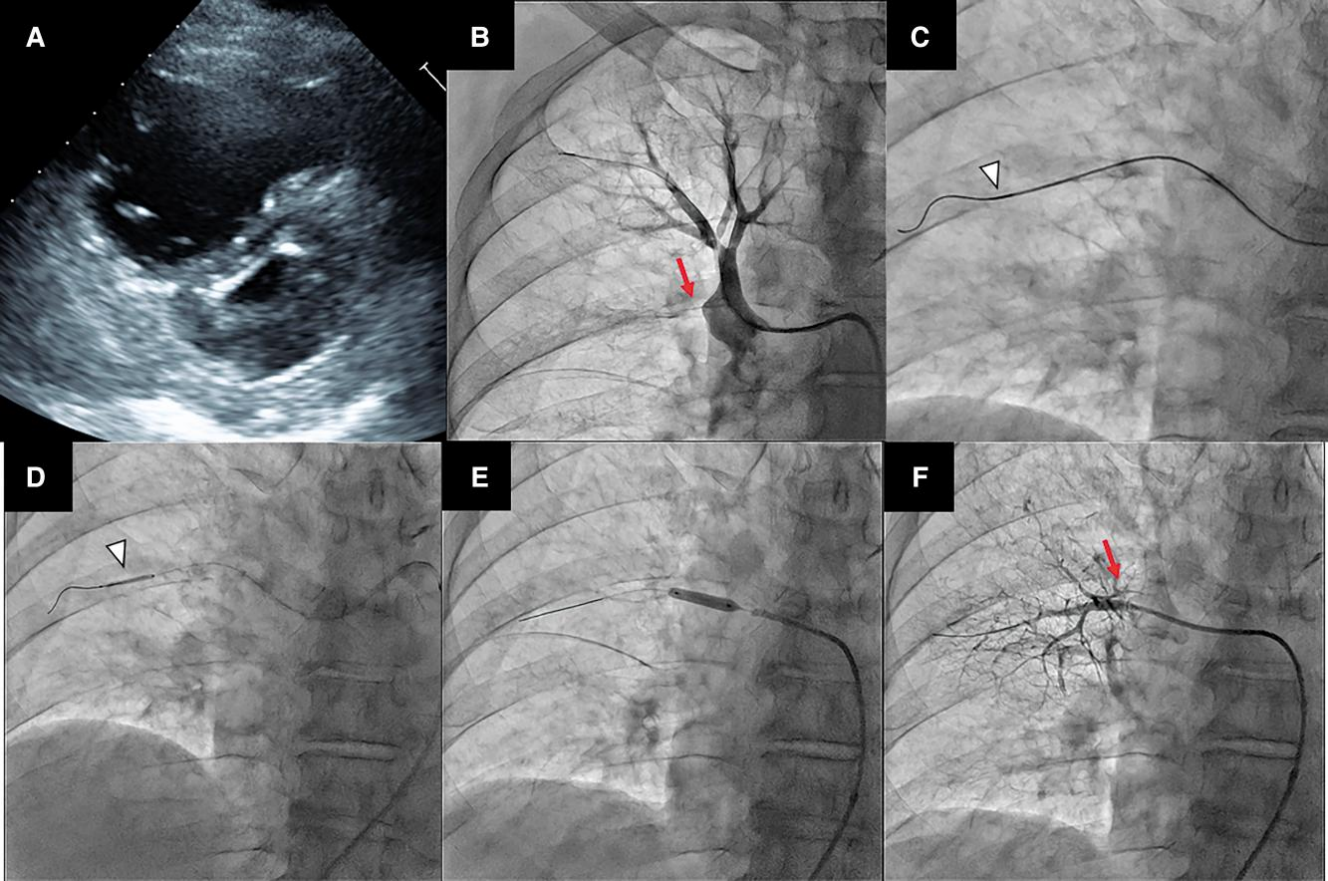
(*A*) Echocardiography revealed a flattened interventricular septum. (*B*) Pulmonary angiography revealed total occlusion lesion in the A3 segment (arrow). (*C*) Corsair enabled passage to total occlusion lesion with the rotational bougie technique (arrowhead). (*D*) Passage and dilation of a 2 mm balloon (arrowhead). (*E*) Passage and dilation of a 4 mm balloon. (*F*) Pulmonary angiography after the procedure revealed good reperfusion (arrow) and pulmonary venous return.

A 77-year-old man had presented with dyspnoea 10 months prior. Acute pulmonary embolism was diagnosed and rivaroxaban was initiated; however, symptoms persisted. Echocardiography revealed a flattened interventricular septum (*Figure 1A*, see supplementary material online, *Video S1*). Lung perfusion scintigraphy revealed multiple wedge-shaped defects. Right heart catheterization revealed a pulmonary arterial pressure (PAP) of 104/30 mmHg, mean pressure of 52 mmHg, and pulmonary vascular resistance of 8.64 Wood units. Pulmonary angiography (PAG) showed total occlusion lesions (TOLs) in the right A3, A4, and A6 segments and subtotal lesions in the bilateral several segments. The patient was diagnosed with chronic thromboembolic pulmonary hypertension (CTEPH). Surgical treatment was proposed but rejected by the patient. After consulting cardiology team, balloon pulmonary angioplasty (BPA) was suggested. Riociguat and selexipag were initiated before BPA. After two BPA sessions for easy-to-treat lesions, the mean PAP improved to 31 mmHg. We performed A3 TOL-targeted BPA for further improvement (*Figure 1B*). We crossed a 0.014-inch wire distally to A3; however, the balloons could not be advanced. We rotationally pushed the Corsair microcatheter (AsahiKASEI, Tokyo, Japan) forward and penetrated the lesion (*Figure 1C*). Subsequently, we advanced the balloons and dilated the lesion with a 2 mm (*Figure 1D*) and 4 mm balloon (*Figure 1E*). PAG revealed good reperfusion and pulmonary venous return (*Figure 1F*, Supplementary material online, *Video S2*) and the patient’s symptom subsided.

Selexipag was reported to be effective for CTEPH.^1^ In Japan, it has been approved for CTEPH since August 2021. The patient’s PAP was significantly high; therefore, we initiated combination therapy of riociguat and selexipag. Surgery is the standard therapy of CTEPH; however, BPA is a possible alternative. Treatment of TOLs is challenging. Kawakami *et al.* reported a success rate of 52%^2^; however, successful treatment of TOLs significantly improves hemodynamics.^3^ Although wire passage through TOLs is feasible, balloon passage is sometimes hindered by substantial thrombosis. Therefore, lesion penetration and route creation using Corsair enables balloon passage. Rotational push improves Corsair passage. This Corsair rotational bougie technique facilitates balloon passage through TOLs in CTEPH.

## Supplementary Material

ytad040_Supplementary_Data

## Data Availability

The data underlying this article are available in the article and in its online Supplementary material.

## Supplementary material

**Consent:** The study was published with the written consent of the patient in line with Committee on Publication Ethics guidelines.

**Funding:** None declared.
